# Auditory-Verbal Music Play Therapy: An Integrated Approach (AVMPT)

**Published:** 2013-10

**Authors:** Sahar Mohammad Esmaeilzadeh, Shahla Sharifi, Hamid Tayarani Niknezhad

**Affiliations:** 1 *Faculty of Linguistics, Ferdowsi University of Mashhad, Mashhad , Iran.*

**Keywords:** Hearing Loss, Hearing-Impaired Children, Music Therapy, Play Therapy

## Abstract

**Introduction:**

Hearing loss occurs when there is a problem with one or more parts of the ear or ears and causes children to have a delay in the language-learning process. Hearing loss affects children's lives and their development. Several approaches have been developed over recent decades to help hearing-impaired children develop language skills. Auditory-verbal therapy (AVT) is one such approach. Recently, researchers have found that music and play have a considerable effect on the communication skills of children, leading to the development of music therapy (MT) and play therapy (PT). There have been several studies which focus on the impact of music on hearing-impaired children. The aim of this article is to review studies conducted in AVT, MT, and PT and their efficacy in hearing-impaired children. Furthermore, the authors aim to introduce an integrated approach of AVT, MT, and PT which facilitates language and communication skills in hearing-impaired children.

**Materials and Methods:**

In this article we review studies of AVT, MT, and PT and their impact on hearing-impaired children. To achieve this goal, we searched databases and journals including Elsevier, Chor Teach, and Military Psychology, for example. We also used reliable websites such as American Choral Directors Association and Joint Committee on Infant Hearing websites. The websites were reviewed and key words in this article used to find appropriate references. Those articles which are related to ours in content were selected.

**Conclusion:**

VT, MT, and PT enhance children’s communication and language skills from an early age. Each method has a meaningful impact on hearing loss, so by integrating them we have a comprehensive method in order to facilitate communication and language learning. To achieve this goal, the article offers methods and techniques to perform AVT and MT integrated with PT leading to an approach which offers all advantages of these three types of therapy.

## Introduction

Hearing loss is the most common birth defect. It exists when there is lack of sensitivity to the sounds normally heard. It has been known for some time that children with hearing loss will pass the usual stages in learning speech language through prompt referral and by using appropriate reinforcing devices (for example, a hearing aid). When children are at younger age and were provided by language instructions in a "critical period of learning speech and language", their performance in level of learning and communication is more acceptable while they are growing up (1).

Sharma (2002) explained that a child's potential to use speech input is likely to deteriorate if listening is not developed during the critical language-learning years (6 months to 4 years). The key feature of the developing auditory system is plasticity which is present at 6 months and decreases with age (2). Plasticity of neurons is more evident in childhood than in adults, which might enable neural viability with electrical stimulation of the implant (3). 

## Materials and Methods

***Impact of hearing loss in children***

Untreated hearing loss in a child has a significant impact on auditory brain develop- ment with serious consequences for speech, language, literacy, academic achievement, and social/emotional development for the child's life term (P4) (4). This impairment also affects social and family relationships (5).

The auditory-verbal approach is the natural outcome of recent advances in knowledge, skills, and technology. Simultaneously, use of new treatment strategies is required to enhance efficiency of these developments. The emergence of advanced hearing-aid technology and the increasing spread of cochlear implantation have made auditory-verbal strategies and techniques invaluable for many hearing-impaired children. Most hearing-impaired children can listen to speech sounds through use of powerful hearing aids and cochlear implant prostheses. The mission of the auditory- verbal approach is to help hearing-impaired children to listen and hear so as to communicate through speech. Pioneers in the auditory-verbal approach, Dorian Pollack, Daniel Ling, and Helen Beebe, introduced it as a set of instructions, directions, and protection for the family (6).

AVT is an educational approach increasingly employed worldwide by the parents and instructors of hearing-impaired children, particularly young children (7). It focuses on developing listening and spoken language through audition, using parents as the child's natural language teachers, with the aim of full inclusion into the mainstream (8).

Auditory stimulation influences the organization and maturation of auditory brain pathways, allowing a child to make meaning from what they hear (9). Consequently, it is essential for early and ongoing auditory and educational intervention to access and develop the auditory brain centers (10).

**Auditory-verbal therapist** **:**

An auditory-verbal therapist is a mentor, psychologist, audiologist, or speech-language pathologist who follows the principles of the auditory-verbal approach. Most auditory-verbal therapists have a degree in one of the above majors, which is indispensable for obtaining a certificate of auditory – verbal approach. Auditory - verbal therapists acquire advanced clinical experience through academic courses at special centers for auditory-verbal therapy or from auditory-verbal experts (11).

***Early detection and intervention***

Early detection and intervention have a great impact on children with hearing loss. According to experienced experts in the field of rehabilitation of hearing-impaired children, it is clear that identification in the early ages is more significant and more effective than any other factors which impact on rehabilitation programs. Studies show that the early detection of hearing loss along with immediate and appropriate intervention (e.g. hearing aid) in children, particularly in the first 6 months of life, can help them to improve language skills (12), speech production (13), and also social and emotional development (5).

There are two kinds of intervention for children with hearing loss, audiological intervention and educational intervention. The latter refers to methods by which children can learn to hear and communicate and the former, as Dornan (2010) mentioned, refers to diagnostic assessment to determine the nature and degree of hearing loss, followed by fitting of one or two hearing devices to allow the child's brain access to auditory stimulation. Digital hearing aids and cochlear implants are the two most common types of hearing technology used these days. However, hearing loss children need to be trained in using these electronic devices.

Educational intervention plays a significant role in the life of hearing-impaired children. These children must develop language in order to communicate, and it is the parents’ responsibility to choose an educational intervention for their children. However, a method of communication should be selected first. Specific communication options are described and considered within a continuum of spoken and visual language by Gravel and O'Gara (2003). They defined "a communi- cation option, mode, modality, or method is the means by which the child and family receive and express language". In communi cation options, there is a range of choices from signed options to listening and spoken language options, and parents choose one to communicate with their hearing-impaired children. Choice depends on a number of factors including the type of information presented to parents and how it is presented, the programs available in the child's area, the personal philosophies of parents and clinicians and how well the child is already communicating at diagnosis (4) (p7).

**Fig 1:**
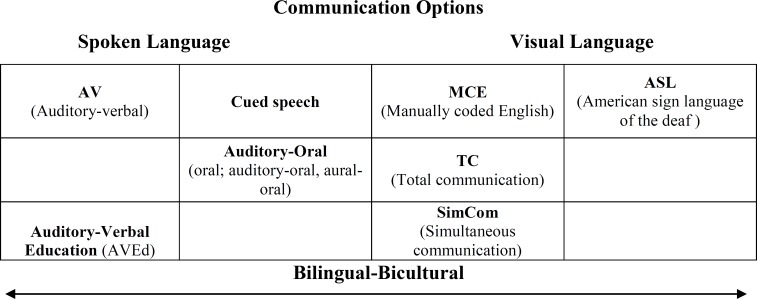
Diagrammatic representation of the communication options showing how each relates broadly to spoken language or visual language. Adapted from Dornan (2010).

The various educational options for children with hearing loss include bilingual/bicultural, total communication, cued speech, auditory-oral and AV approaches. Bilingual/bicultural programs focus on education through two languages, such as sign language, the language of the deaf, and English, where English is taught as a second language through reading, writing, or sign. Total communication is the combined use of aural, manual, and oral modalities in communicating with and teaching individuals with hearing loss. Cued speech is a visual communication system using eight hand shapes in four different locations, in combination with the natural mouth movements of speech, to make all the sounds of the spoken language look different. The auditory-oral approach teaches children with hearing loss to use their residual hearing in combination with speech reading and other cues to better comprehend and use spoken language. With an auditory-oral approach, the typical delivery model is a classroom setting with groups of children with hearing loss. The education option of AVT is the application of techniques, strategies, conditions, and procedures that promote acquisition of spoken language through listening, and is a one-on-one parent-centered approach. This approach focuses on developing listening and spoken language through audition, using parents as the child's natural language teachers. (4) (P16).

There are several factors which affect parents’ decision in selecting one of these options such as age of identification and intervention, use of hearing aids/cochlear implant, speech intelligibility, presence of additional disabilities, etc**. **However, none of these options are optimal for hearing-impaired children. The ultimate goal in choosing any communication option is to ensure that hearing-impaired children and their families become language proficient and fluent communicators (14).

AVT can help hearing-impaired children to acquire spoken language through listening. Parents and instructors have a significant role in AVT. Auditory-verbal therapy was first described by Doreen Pollack (1970), then Auditory-Verbal International Inc. in 1993 used this description and developed the 10 principals of AVT. Later in 2007, the Alexander Graham Bell Academy for Listening and Spoken Language confirmed these principals, which are to: 

1. Promote early diagnosis of hearing loss in newborns, infants, toddlers, and children followed by immediate audiologic management and AVT. 

2. Recommend immediate assessment and use of appropriate, state-of-the-art hearing technology to obtain maximum benefits of auditory stimulation. 

3. Guide and coach parents to help their child use hearing as the primary sensory modality in developing spoken language without the use of sign language or emphasis on lip-reading. 

4. Guide and coach parents to become the primary facilitators of their child's listening and spoken language development through active, consistent participation in individualized AVT. 

5. Guide and coach parents to create environments that support listening for the acquisition of spoken language throughout the child's daily activities. 

6. Guide and coach parents to help their child integrate listening and spoken language into all aspects of the child's life. 

7. Guide and coach parents to use natural developmental patterns of audition, speech, language, cognition and communication. 

8. Guide and coach parents to help their child to self-monitor spoken language through listening. 

9. Administer ongoing formal and informal diagnostic assessments to develop individualized auditory-verbal treatment plans, to monitor progress and to evaluate the effectiveness of the plans for the child and family. 

10. Promote education in regular classrooms with peers who have typical hearing and with appropriate support services from early childhood onwards. 

The AG Bell Academy has introduced a new option which is auditory-verbal education (AVEd). The main differences between AVT and (AVEd) are that in AVEd, it is not necessary for parents to be physically present and children can be taught in a group or as an individual. However, in AVT the parent is the main focus of the intervention and he/she is always present with the child in AVT sessions, which are always individual. (4)

The aim of AVT also is to enable children with hearing loss to grow up in regular learning and living environment (15). Several studies describe that AVT is a practical method for teaching deaf and hard-of-hearing children in order to help them live in the hearing world (16,17).

Dornan (2010) also concluded from his longitudinal study that AVT is an effective education approach. He suggested that the AVT group made significant progress in language, receptive vocabulary, and speech skills. Also, Rhoades and Chisolm (2000) reported in their study that AVT has an impact on developing both receptive and expressive language abilities, and that the gap between chronological age and language age was closed by intensive auditory-verbal intervention.(16)

As a result, it is expected that children who receive AVT in their preschool years will join mainstream school classes in their school age.

Some new studies also claim that music has an impact on the language learning of hearing-impaired children. These studies introduce a method in which music is used to calm or even cure patients. In fact, there is a growing field of health care known as music therapy (MT), which uses music to heal. Here we briefly explain it.

***Music therapy and*** ***music processing***

From ancient times, music and singing have been a meaningful part of human experiences and there are many stories, poems, and literature as well as historical texts about mothers who sing for their children. Music and memory are highly interdependent. Our experiences show that stories and poems for children remain in the mind for many years. Although complete hearing loss can be a major obstacle to the enjoyment of music, most hearing-impaired children who have residual hearing can enjoy music or even sing by intensifying it(1).

Music has been used in medicine for thousands of years. Ancient Greek philosophers believed that both the body and the soul could be healed by music. The history of music as a way of healing goes back to the time of Aristotle and Plato.  However, in the twentieth century, the formal idea of using music refers to comfort the victims of the First World War. Afterward in 1944 the world's first MT program at the University of Michigan began. 

Aldridge (1994) explained that there are two principal methods MT: active and passive MT. In active MT, the patients themselves play music instruments or sing with the therapist, while in passive MT the patients listen to music (18). Music therapy is used in many settings, including schools, preschools, rehabilitation centers, hospitals, hospices, nursing homes, community centers, and sometimes even in the home.

Bruscia (1998, p.20) has defined MT in his book by stating that: “Music therapy is a systematic process of intervention wherein the therapist helps the client to promote health, using music experiences and the relationships that develop through them as dynamic forces of change.” (19)

Gold and colleagues (2004) stated that MT has a positive effect on psychopathology of children and adolescents. The authors concluded that MT is an effective intervention for these children with psychopathology (20).

Some research studies have shown that music has a profound effect on our body and psyche. Several studies have been conducted to identify the effect of MT on depression (21), emotional exploration (22), anxiety and anger (23), and mental disorders (24). In all these studies, results show that MT has a positive effect on reducing depression, stress, and anxiety in patients by managing some of their symptoms, and expressing feelings relating to their experiences. Furthermore, it can help mental disordered patients to participate successfully in social activities. So MT has a long history and music has been used as a means of communication and healing since the beginning of mankind.

Although interest in music and the mind go back to the time of Plato, the neuroscientific study of music is rather a new field. Many studies have been conducted on the process of music in the brain. 

Koelsch et al. (2005) has investigated the functional neuroanatomy of music perception with functional magnetic resonance imaging (fMRI) in children and adults. They found that in both adults and children, musical training was correlated with stronger activations in the frontal operculum and the anterior portion of the superior temporal gyrus. They classified the activated cerebral structure into four cortical networks:" (a) a network comprising IFLC (along with the anterior STG) and vlPMC involved in the processing of musical structure, (b) a network comprising posterior temporal areas (BA 21, BA 22p, and BA 37) involved in the processing of musical meaning, (c) a network comprising SMG and pre-frontal cortex (BA 45) related to working memory, and (d) a network comprising OFLC (BA 47), and anterior insula, possibly involved in emotional aspects of music processing" (25). 

Sammler et al. (2010) have described that music, like language, has syntax. Music has a set of rules (e.g., harmonic and morphosyntactic principles) by which we can combine notes or chords to produce a piece of music. Some areas in the brain are responsible for processing syntax in language. These areas also play a functional role in the processing of musical syntax (26). Musical abilities which relate to the left hemisphere share some properties like duration, temporal order, simultaneity, and rhythm with speech, and would therefore take a greater role when the sequential and analytic aspects of music are more important; however the right hemisphere is very important in many other aspects including perception of loudness, timber, intonation, and the expression of emotion. In a normal music situation, perception depends on the synthesis of pitches and rhythms; both processes are involved, not in terms of the specialization of one hemisphere that is "dominant" for music, but as an interaction of both hemispheres, each operating according to its own specialization, in the complex process of music perception (27). Clinical and experimental evidence suggests that the left hemisphere of the brain is specialized for speech activity and the right hemisphere is specialized for many non-linguistic functions. The human brain is very complex in the way it perceives information that it receives through sensory input. The two cerebral hemispheres perform different functions; the left hemisphere processes information that requires analysis or some type of language to comprehend, and the right hemisphere deals with the symbolic, non-verbal and emotional portion of what we call reality. By arousing different parts of the brain, music can be taken in to consideration as an effective therapeutic or mood-altering tool. Pitch, rhythm, meter, and timber – the distinctive features of music – are processed in various parts of the brain ranging from the pre-frontal cortex to the hippocampus to the parietal lobe. Rhythm and pitch are primarily left brain hemisphere functions, while timber and melody are processed primarily in the right hemisphere. Meter is processed in both hemispheres. (28)

***Right brain or left brain or both***

For years it has been thought that like the left hemisphere which is responsible for language, the right hemisphere is responsible for music. However, some research suggests that both the right and left hemispheres of the brain are responsible for processing music. One hemisphere should not be regarded as “dominant” for music. There is an interaction between these hemispheres. (27). While the right brain does process rhythm patterns, timber, harmonic function and emotional responses to music, the left brain is also involved. Analytical and formal structures are processed in the left brain, as well as stylistic and artistic elements. In fact, active musical participation, perhaps more than any other activity, engages more parts of the brain and encourages the two hemispheres to work together effectively and efficiently. (29)(p3) Both the right and left hemispheres are involved because while one codifies lyrics the other tackles melody.

***Music perception in children with cochlear implants***

Music forms part of our daily life and also is part of our culture and leisure. But, deafness deprives patients of all sorts of music. Music does have a number of objectively identifiable features such as rhythm, timber, pitch, etc. We could say that music perception is the perception of these features. 

Although, by the advent of cochlear implants technology, deaf and hard of hearing children can increase language perception, the perception of music is not resolved by current cochlear implants (30). Cochlear implants technology help children to improve language perception, production and comprehension depending on the extent of their hearing loss and other variables (31). So the information transmitted by this electronic device is sufficient for language perception but it is not adequate for music perception. 

Many researches have investigated music perception in children with cochlear implants. They argued that these children have problem on distinguishing some features of music like rhythm and timber (32,33). Drennan (2009) referred to issues concerning music perception with cochlear implants. He describes that by improving children's overall hearing and speech understanding in quiet and in noise; we can help them to improve their music perception which has a significant impact on their life. He also referred to the advantage of cochlear implants, which is delivering of rhythm. These devices, however, have some shortages. For example, they cannot hear exact pitches or tones of a chord as normal hearing listeners do (34).

Lassaletta et al. (2008) mentioned that the perception of simple musical patterns (rhythm or tone) is much easier for these children than the perception of complex patterns. They also emphasized that the quality of music sound through the cochlear implant affects post-operative quality of life (30).

Overall, by improving cochlear implant devices, music perception could be possible for hearing-impaired children and through MT, hearing loss children can improve their communication and environmental awareness.

***Play therapy and hearing-impaired children***

Play can be used as a medium to help therapists interact with children and help them express their feelings and emotions. We can provide a way for children to express their experiences and feelings through a natural, self-guided, self-healing process. As children’s experiences and knowledge are often communicated through play, it becomes an important opportunity for them to know and accept themselves and others. Although games play a significant role in child development and many studies have been conducted in this aea, there are a few definitions of the word game. The use of play in therapy was first explained by the pioneers of Child Psychotherapy such as Anna Freud who believed in positive effects of play on child development (1928).

There is a central proposition among child psychotherapists that play transmits and communicates the child's unconscious experiences, desires, thoughts, and emotions. Several researchers have elucidated that use of play therapy (PT) in the treatment of young patients can help them to handle their problems such as disruptive school behavior, parenting stress, and other issues (35,36). Bratton and Ray (2000) also imply that some behavioral problems can be treated by PT; furthermore it has a favorable impact on improving children’s self-concepts, social skills, and anxiety symptoms (36). PT is an effective intervention for childhood difficulties such as conduct disorder, fear, speech and language difficulties, and depression (37). PT treatment programs have been used as the primary intervention in treating many disorders such as grief, fear, and anxiety, dissociation and schizophrenia, autism, reading and speech difficulties; also it improves self-concepts and self-esteem in young children. (38). Thus, according to the conspicuous role of play in children psychological development, some therapists use PT as an intervention in the treatment of their young patients. The Association for Play Therapy (APT) has defined PT as "a systematic use of a theoretical model to establish an interpersonal process wherein trained play therapists use the therapeutic powers of play to help clients prevent or resolve psychosocial difficulties and achieve optimal growth and development". PT helps children to simulate, so that they can cope with their problems that they have not had the chance to sort out properly. Since play requires active involvement, instructional playing is a way for increasing the performance and motivation of learners by creating excitement and enjoyment (39). Some researchers found that instructional games and plays have a positive effect on improving learning ability in children (40)**. **

Play therapists are trained in teaching children. They are acquainted with how to behave and treat children in a way that encourages them to participate in therapy. They also know how communicate with children about their feelings and thoughts. The role of the play therapist is to offer each child a safe and warm space where they feel calm and able to be themselves without any anxiety. 

Although play has a significant role in children's life, even hearing-impaired children's, few articles have been written on PT in hearing-impaired children. Casby and McCormack (1985) have investigated the relationship between symbolic play and language performance for young hearing-impaired children. They concluded that there is a strong positive relationship between the variables of symbolic play and early communication development (41)**.** For the child, PT can facilitate the expression of their feelings and fears through the natural activity of play. In summary, through PT children can improve their behavior, increase their self-concepts as well as their self-esteem, understand how to cope with their feelings, and improve their difficulties in speech. PT is regarded as a viable and important tool in working with all children even hearing-impaired ones. It allows children to express their thoughts and feelings through language of play. For deaf and hard of hearing children, it is practical because play can be more visual than language-based communication. So we can use this kind of intervention-play therapy- along with other techniques such as AVT and MT in teaching hearing-impaired children.

## Discussion

The concept of integrating some of the basic principles of AVT, PT and MT provides a basis for a new combined therapy modality best called auditory-Verbal Music Play Therapy. In AVMPT, the teacher or therapist use the principals of AVT to teach language items to hearing-impaired children by musical linguistic plays. In this approach a child is free to play out his feelings. Musical instruments allow for an exploration of feelings, as do traditional PT materials. Hearing-impaired children typically soon involve themselves in the process of therapy which consists of AVT principals, play, and music. To teach an educational term to hearing-impaired children like teaching jobs, we try to create a play or game representing that job which we want children to learn. By giving an example we shed some light on the AVMP approach. 

In this approach if the instructor is going to teach the word "teacher", she should do it in the form of a play. First she has to prepare an appropriate physical space which consists of classroom, hearing-impaired children, teacher and/or therapist. Then, by giving a proper role to the child, parents and the teacher, the therapist also provides an appropriate acoustic space without noise, to teach the term.

According to strategies like use of a singsong voice, modeling correct language, repetition, proximity to the microphone, etc. (42) we try to teach the target term –which is the word “teacher”- by repeating the word in various sentences and also in a musical and rhythmic way as well as in poetic form. We can also use dance and appropriate play representing the term. Also, by giving the role of the teacher to the mother, the role of student to the child and the role of the classmate to the father, and taking advantage of the other techniques and strategies for developing listening skills like audition first and turn taking, we can stimulate the child's speech. In addition, the therapist and parents should try to accept the child's speech and encourage him/her to learn and produce new sentences. Also they should complete their speech. For example if the child says "good teacher", the parents or therapist should complete the sentences while encouraging the child. They can say "the teacher is good"; "the teacher likes students"; or "the teacher teaches us". These sentences should be produced in a rhythmic form and also accompanied by related verbal activities. If these verbal activities go together with physical activities and stimulate the motivation and emotions of the child, other brain systems along with auditory areas like the limbic area, will be involved. Conscious experiencing of a sensory stimulus activates both the sensory and motor areas of the brain (43). These integrated activities cause the improvement of neuroplasticity in the brain and also stimulate the learning and memory area of the brain. Therefore, by using an interesting and fun play containing different auditory-verbal techniques, the child can learn the target instructional term more effectively, and subsequently use that term in communicative activities.

## Conclusions

AVT, MT, and PT have a significant role in creating a normal environment for hearing-impaired children by helping them to reduce the impact of their hearing imperfection and giving treatment for both children and their family. So the concept of integrating some of the basic principles of these three approaches provides a basis for a new combined therapy which comprises all practical and beneficial aspects of AVT, MT, and PT. So the use of AVMP therapy in a hearing-impaired children classroom can be a potentially powerful pedagogic tool. Therefore we can use this new approach to facilitate communication and language learning in hearing-impaired children. This article is intended solely to introduce this integrated method, so further study of the applicable implications of this new integrated therapy is recommended.
